# A scoping review of the relationship between autistic traits and eating disorders: exploring the secondary impact of eating disorders and co-occurring psychiatric diagnoses

**DOI:** 10.3389/fpsyt.2026.1837078

**Published:** 2026-05-11

**Authors:** Jess Kerr-Gaffney, Emy Nimbley, Amelia Austin, Karri Gillespie-Smith, Helen Sharpe, Fiona Duffy

**Affiliations:** 1 Institute of Psychiatry, Psychology and Neuroscience, Kings College London, London, United Kingdom; 2 Department of Clinical & Health Psychology, University of Edinburgh, Edinburgh, United Kingdom; 3 School of Health and Wellbeing, University of Glasgow, Glasglow, United Kingdom; 4 Department of Psychology, School of Health and Life Sciences, Glasgow Caledonian University, Glasgow, United Kingdom; 5 NHS Lothian Child and Adolescent Mental Health Service, Edinburgh, United Kingdom

**Keywords:** anorexia nervosa, autism, avoidant/restrictive food intake disorder, binge eating disorder, bulimia nervosa, eating disorders, neurodiversity

## Abstract

**Objective:**

Research has highlighted co-occurrence and phenotypic overlap between autism and eating disorders (EDs), however the origin of this overlap is uncertain. The aim of this scoping review was to assess existing evidence on the role of acute illness effects and co-occurring mental health difficulties in the relationship between autism and EDs.

**Methods:**

The review was conducted following the PRISMA extension for scoping reviews. Electronic databases (PsycINFO, PubMed, Embase and Web of Science) and grey literature (ProQuest Dissertation and Theses) were searched up until 11th January 2026 for quantitative, qualitative, and mixed-methods empirical studies using autism and ED search terms.

**Results:**

Longitudinal and qualitative evidence reporting Autistic traits in childhood, a lack of association between BMI and Autistic traits, and a link between autism and EDs not typically associated with low body weight suggests that the association between autism and EDs is not solely due to acute illness effects. High rates of additional mental health problems in those with EDs were not found to fully account for co-occurring autism or Autistic traits.

**Discussion:**

Areas for further research include ED populations other than anorexia nervosa (AN), and EDs in Autistic individuals rather than trait-based research. The review highlights the need for early identification of autism and support for Autistic young people, as well as improved training and autism-specific support in ED and other mental health services.

**Systematic review registration:**

Open Science Framework, https://osf.io/t5fwg.

## Introduction

Eating disorders (EDs) are severe psychiatric conditions associated with substantial medical and psychological morbidity and elevated mortality (1). Recent systematic reviews and meta-analyses demonstrate a higher prevalence of autism and Autistic traits among individuals with EDs, particularly in anorexia nervosa (AN) (2) and avoidant/restrictive food intake disorder (ARFID) (3) compared to peers without EDs. Individuals with EDs frequently exhibit cognitive, behavioural, and social characteristics and symptoms that overlap with core features of autism, including differences in social communication, executive functioning, sensor sensitivities and emotion processing (4–6). This phenotypic overlap raises important questions regarding the interpretation of elevated rates of autism and Autistic traits in individuals with EDs.

There is ongoing debate amongst the literature regarding whether increased prevalence of autism and Autistic traits in EDs reflects a true overlap, i.e., ‘trait’ effects, or whether they may be consequences of the ED and not present before ED onset, i.e., ‘state’ effects (7–9). On the one hand, theoretical models have proposed that Autistic people may be at increased risk of EDs via direct pathways (e.g., food-related sensory aversions or intense, focussed interests on eating or exercise resulting in restrictive eating) and indirect pathways (e.g., autism-related difficulties such as social exclusion or intolerance of uncertainty leading to emotional distress, and disordered eating is then used to reduce this distress) (10). Conversely, concerning the ‘state’ argument, some have suggested that elevated Autistic traits are secondary cognitive or behavioural consequences of EDs (e.g., starvation, social changes); that the overlap is better explained by co-occurring mental health conditions, such as obsessive-compulsive disorder (OCD) or depression; or that it reflects methodological or measurement limitations of ED research on Autistic traits and autism diagnosis.

Some argue that the impact of starvation observed in EDs mimic Autistic traits such as cognitive rigidity, repetitive behaviour or social differences, and suggest that elevated Autistic traits are therefore not reflective of true autism (8, 9). Support for this argument comes from studies reporting reductions in Autistic traits following weight restoration in individuals with AN (11–13). However, numerous studies have reported that elevated Autistic traits persist following weight recovery (14–16). Further studies that refute this line of argument come from qualitative and longitudinal evidence that report the presence of Autistic traits in childhood and therefore proceeding the onset and potential impact of the ED (10, 17, 18), and studies that report elevated Autistic traits in non-restrictive EDs such as binge eating disorder (BED) and bulimia nervosa (BN) (19). Also of note, studies that have reported a reduction following weight restoration in Autistic traits still report a clinically significant level of Autistic traits in a notable number of their sample [e.g., ~25% in Nuyttens et al. (13)].

Others have highlighted the possibility that common co-occurring mental health conditions may better explain the association between autism and EDs. For example, symptoms of co-occurring conditions such as anxiety, depression, or OCD could inflate scores on autism measures (e.g., 20). Alternatively, the observed association could reflect shared co-occurrence between autism, EDs, and other mental health problems. High rates of co-occurring conditions are seen in Autistic individuals and those with EDs (21, 22). These co-occurring conditions may mediate the relationship between autism and EDs, such that Autistic individuals are at greater risk of developing conditions such as depression, which in turn increases the risk of developing an ED (23). This line of argument does not seek to refute that there is a relationship between autism and EDs, but instead that this relationship may not be specific to autism itself.

A final area of debate surrounding the overlap between autism and EDs is regarding the quality of the research itself. For example, many studies lack control or comparison groups, making it difficult to ascertain whether samples with EDs differ from unaffected controls or other psychiatric groups (7). Further, most research has used cross-sectional, self-report measures of Autistic traits rather than diagnostic approaches, emphasising a need for high quality longitudinal research (7, 24). While this may be a pertinent point in the context of research design, it is important to highlight evidence to suggest that similar clinical characteristics and treatment outcomes are reported in individuals with EDs with high Autistic traits versus those with an autism diagnosis (25, 26).

Thus, despite this significant increase in autism and ED research (27), many remain cautious of interpreting this overlap as a true aetiological association. There is conflicting evidence amongst the literature and there remains an urgent need to scope the existing evidence base, map this evidence in line with concerns articulated in the ED field, and integrate these findings into a singular, cohesive presentation of the evidence-base to date. This scoping exercise will also allow for the identification of possible gaps where our understanding is limited and highlight future research can do to advance our clinical understanding. The current scoping review therefore aimed to ask the following questions:

1. Can the overlap between autism and EDs be better explained as a secondary consequence of EDs (e.g., starvation, social withdrawal)?
2. Can the overlap between autism and EDs be better explained by co-occurring mental health conditions?

## Methods

The review was conducted in accordance with the Joanna Briggs Institute guidelines for scoping reviews (28) and the PRISMA extension for scoping reviews (29). An a-priori protocol was pre-registered with the Open Science Framework (https://osf.io/t5fwg).

### Eligibility criteria

In line with review aims, included studies (see **Table 1**) were required to explore whether elevated rates of Autistic traits or autism reported in ED populations were the results of (a) the secondary impacts of EDs (e.g., impact of starvation, behavioural consequences such as social withdrawal or emotional avoidance) and/or (b) better explained by the presence of co-occurring conditions (e.g., depression, anxiety). See **Table 1** for a summary for inclusion and exclusion criteria.

**Table 1:** Inclusion and exclusion criteria for studies.

Domain	Inclusion criteria	Exclusion criteria
Publication type	Peer review journal articles, conference abstracts, dissertations	Practice guidelines, letters, commentaries, editorials, opinion or reflection pieces, newsletters
Language	English	Non-English language (without capacity to translate)
Objectives	Explore potential for elevated Autistic traits in eating disorder populations to be a secondary consequences of the eating disorder or better explained by co-occurring psychological conditions	No focus on interpreting the interaction between Autistic traits and the secondary consequences of eating disorders or other co-occurring psychological conditions
Methods	Quantitative, qualitative, mixed-methods empirical studies	Narrative reviews, systematic or scoping reviews, meta-analyses
Design	Any study design (including but not limited to longitudinal, case reports, experimental or quasi-experimental, interviews)	
Sample	Any human subjects	

### Search strategy

Four electronic databases (PsycINFO, PubMed, Embase and Web of Science) and grey literature (ProQuest Dissertation and Theses) were searched for studies up until 11th January 2026. Following the screening and selection process, citation chaining was conducted on full-text papers. Search terms included autis* OR ASD OR Asperger* AND eating disorder OR anorexi* OR bulimi* OR “binge eating*” OR ARFID OR “avoidant and restrictive feeding intake disorder*”.

### Study screening and selection

Initial search results were imported into Covidence (https://www.covidence.org/). Duplicates were removed before two reviewers independently screened at title and abstract using the eligibility criteria (EN, FD). Full-text articles were then screened by one reviewer (EN) with a second reviewer (FD) independently screening 30%. All studies that did not meet eligibility criteria from full-text screening were excluded and reasons for exclusion were documented. Any discrepancies between reviewers were resolved by mutual consent, or with a third reviewer in instances whereby consensus could not be reached.

### Data extraction

Study data was extracted using a pre-piloted data extraction form, based on the Covidence data extraction template and adapted to suit study aims, methods and population. Data extraction was conducted by one reviewer (JKG), and a second reviewer (AA) independently extracted 30%. Extracted data was as follows: General (author, year; title), Methods (aims and objectives, study design, methods and methodology), Participants (inclusion and exclusion criteria, recruitment, participants, groups), Measures (autism status, autism measure(s), eating behaviours status, eating disorder measure(s), other relevant measures), Analysis (including sample size calculation, statistical analyses, control/confounding variables, missing data), Results (population characteristics, outcomes, summary of study findings). Inconsistencies were resolved via consensus discussions.

### Result synthesis

Results were narratively synthesised in two sections corresponding to review aims (1): Secondary impact of EDs; and (2) Impact of co-occurring conditions. Within the former section, results were further synthesised in the following sections: Autistic traits or diagnoses in childhood/before ED; stability of Autistic traits or diagnoses over time, including before and after weight restoration; Autistic traits or diagnoses in acute and recovered AN; autism and other EDs; associations with BMI; and genetics of autism and EDs.

## Results

### Study selection

**Figure 1:**
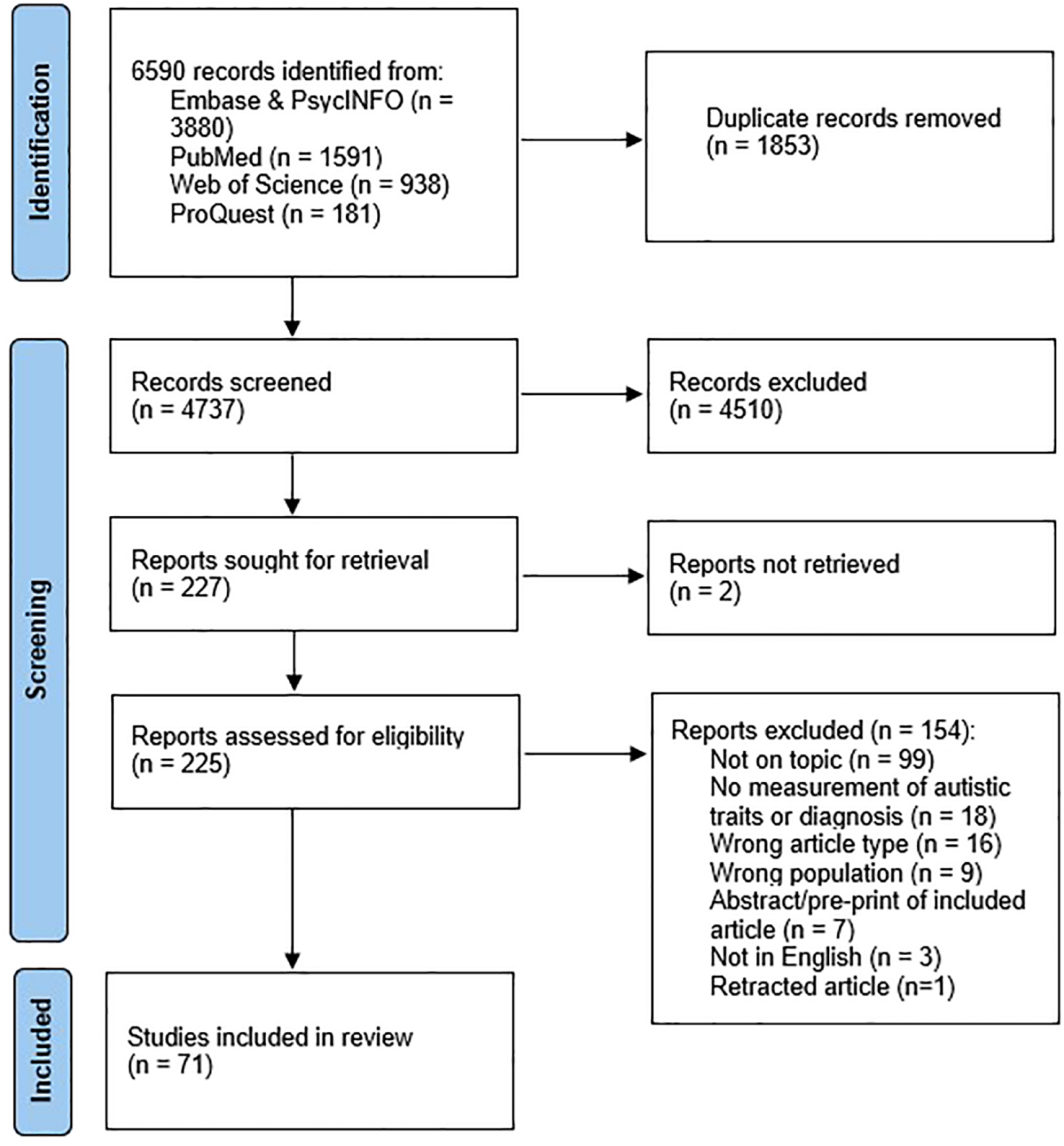
Study selection flow diagram.

Results of the study selection process are shown in **Figure 1**. Seventy-one articles met inclusion criteria and were included in the review.

### Study characteristics and results

Study characteristics are presented in **Table 2**. Most studies were quantitative (n = 59), with either observational, case-control, cohort, clinical audit/service evaluation, survey, or randomised controlled trial designs. The remainder were qualitative (n = 8) or case studies or series (n = 4). The total number of participants in each study ranged from 1 (90, 93) to 1,724,189 (56). Most study samples comprised individuals with EDs and/or were Autistic, with or without a control group (N = 57) and 11 were general population samples often including individuals with EDs and/or were Autistic. Four studies included groups based on other diagnoses [i.e., schizophrenia, attention-deficit/hyperactivity disorder (ADHD), intellectual disability (ID)/intellectual developmental disorder (IDD), or borderline personality disorder (BPD)] (35, 39, 52, 81), four included parents or carers of Autistic individuals with EDs (10, 83, 84, 87), and two overlapping studies included healthcare professionals with experience of working with Autistic people with EDs (10, 84). Twenty-nine studies included exclusively female participants and one study included exclusively males, whilst most studies included a mix of genders. Mean/median age of participants ranged from 2.5 (73) to 55 years (87). Only 15 studies reported race or ethnicity data for participants, with the proportion of White participants ranging from 40% (89) to 97% (49). Regarding socioeconomic indicators, 18 studies reported participants’ or their parents’ education levels, whilst only one study reported income.

**Table 2:** Characteristics of included studies.

Study	Design	Objective	Participants	% female	Mean age (yrs)	Ethnicity	Education	Income	Relevant findings
Quantitative									
Acikel & Cikili (94)	Cross-sectional survey	Investigate the relationship between autism and AN, and the effects of obsessive-compulsive traits in a non-clinical sample	290 university students	50	21.1	NR	All university students	NR	Relationship between AQ-50 and EAT-40 became non-significant when controlling for MOCI.
Anckarsater et al (62)^a^	Longitudinal case-control	Characterise personality disorders, Autistic traits, neurocognition, sociocommunicative difficulties, and outcome in AN with and without autism	51 AN	94	32	NR	NR	NR	Substantial agreement in autism diagnoses across study time points, but only fair agreement with premorbid diagnosis. No difference in comorbidities between AN and autism+AN groups. Lower socio-cognitive performance in autism+AN versus controls.
			52 controls	NR		NR	NR	NR	
Archibald et al (70)	Cross-sectional observational	Examine whether the AQ-10 discriminates between Autistic and non-Autistic young people with possible ARFID	246 possible ARFID	50	11.2	NR	NR	NR	35% had an autism diagnosis prior to ARFID assessment. A further 40% were queried by the ARFID team to be Autistic, whilst 25% were not.
Barnett et al (92)	Cross-sectional survey	Examine potential moderators (sex, anxiety, and depression) of the association between Autistic traits and disordered eating	691 general population	61	23.6	NR	NR	NR	AQ-50 significantly predicted EAT-26 after controlling for anxiety, depression, sex, age, and BMI.
Bentz et al (67)	Cross-sectional case-control	Investigate social function and cognition in young females with first episode AN and those recovered from AN	43 AN	100	16.1	NR	16.1 years^1^	NR	ADOS-2 scores did not differ in acute and rec AN, scored significantly higher than controls. ADOS-2 scores not associated with BMI, anxiety or depression. Social cognition was reduced in rec AN, whilst adaptive social behaviour was reduced in acute AN, compared with controls.
			28 rec AN	100	18.4	NR	14.8 years	NR	
			41 controls	100	17.7	NR	15.3 years	NR	
Bertelli et al (33)	Cross-sectional observational	Investigate prevalence of EDs, FEDS, and associated symptoms in adults with ID/IDD with and without autism.	122 Autistic + ID/IDD	42	41.5	NR	NR	NR	Co-occurrence of ID/IDD and autism was associated with higher prevalence of BN, pica, food refusal, and food selectivity. No difference in BMI or AN or BED prevalence between groups.
			84 ID/IDD	44	42.5	NR	NR	NR	
Boltri et al (85)	Cross-sectional observational	Study prevalence of Autistic traits and cognitive and nutritional correlates in malnourished inpatients with AN.	33 AN	91	25.5	NR	NR	NR	EDI-2 scores were significantly correlated with AQ-50 scores, D-Flex-R, and GSQ, whilst controlling for age and BMI. BMI and illness duration were not associated with EDI-2 or AQ-50 scores.
Brede (43)^b^	Cross-sectional observational	Compare characteristics of Autistic women with and without REDs, women with REDs with low Autistic traits, and women with REDs with high Autistic traits but no autism diagnosis.	47 Autistic	92	38.9	83% White, 4% Black Caribbean, 8% Mixed Background, 4% Other	No qualifications 4%, A level/ foundation 15%, Bachelor's 45%, Master's/ PhD 36%	NR	RAADS-14 score and BMI were positively correlated in the Autistic group only. Those with a RED + high Autistic traits showed a similar pattern of Autistic characteristics, including childhood traits, to diagnosed Autistic participants, controlling for age, depression, anxiety, and social anxiety.
			51 Autistic + RED	88	30.9	90% White, 4% Black Caribbean, 4% Indian, 2% Chinese	No qualifications 10%, A level/ foundation 51%, Bachelor's 18%, Master's/ PhD 22%	NR	
			76 RED + low Autistic traits	100	29.7	96% White, 4% Mixed background	No qualifications 9%, A level/ foundation 42%, Bachelor's 33%, Master's/ PhD 16%	NR	
			36 RED + high Autistic traits	100	30.7	97% White, 3% Mixed Background	No qualifications 11%, A level/ foundation 39%, Bachelor's 27%, Master's/ PhD 22%	NR	
Brede et al (25)^b^	Cross-sectional observational	Compare characteristics of Autistic women with and without REDs, women with REDs with low Autistic traits, and women with REDs with high Autistic traits but no autism diagnosis, and test whether BMI is associated with Autistic traits.	69 Autistic	93	39.6	84% White, 16% other	No qualifications 4%, A level/ foundation 19%, Bachelor's 44%, Master's/ PhD 33%	NR	No significant correlation between Autistic traits and BMI in any group. Those with a RED + high Autistic traits showed a similar pattern of Autistic characteristics, including childhood traits, to diagnosed Autistic participants, controlling for age.
			57 Autistic + RED	88	31.5	91% White, 9% other	No qualifications 12%, A level/ foundation 46%, Bachelor's 19%, Master's/ PhD 23%	NR	
			80 RED + low Autistic traits	100	29.8	96% White, 4% other	No qualifications 9%, A level/ foundation 42%, Bachelor's 35%, Master's/ PhD 16%	NR	
			38 RED + high Autistic traits	100	30.3	97% White, 3% other	No qualifications 11%, A level/ foundation 42%, Bachelor's 26%, Master's/ PhD 21%	NR	
Calderoni et al (86)	Cross-sectional case-control	Explore Autistic traits and internalising symptoms in adolescents with AN-R	25 AN	100	14.3	NR	NR	NR	No correlation between BMI and AQ-50. AN and controls with internalising symptoms scored similarly on the AQ-50, higher than controls without internalising symptoms.
			170 controls	100	15	NR	NR	NR	
Carter Leno et al (17)	Longitudinal cohort	Test whether the association between EDs and autism is in part driven by fussy eating	8982 general population	NR	6.8 - 14.1^2^	NR	NR	NR	SCDC scores at age 7 were associated with ED behaviours at age 14, this association was partially mediated by fussy eating.
Carpita et al (34)	Cross-sectional observational	Examine associations among ED, autism, and trauma-related symptoms in people with BPD	29 BPD	93	33.3	NR	NR	NR	BPD + Autistic traits had higher EDI-2 and trauma symptoms than BPD only. Trauma symptoms partially mediated the effect of AdAS on EDI-2.
			44 BPD + Autistic traits	84	29.3	NR	NR	NR	
Christiansen et al (54)	Longitudinal cohort	Investigate diagnostic, familial, and genetic associations of EDs with ADHD and autism.	1671823 general population	49	NR	NR	NR	NR	Those with EDs had increased risk of a subsequent ADHD or autism diagnosis, and people with ADHD or autism had increased risk of an ED, partly mediated by intermediate mood or anxiety disorders. Co-aggregation of ADHD or autism within families. AN had higher autism PGS than controls, Autistic individuals did not differ from controls in AN PGS.
Dell'Osso et al (71)	Cross-sectional case-control	Assess Autistic traits in AN, BN, and BED.	46 AN-R	98	29.3	NR	13.7 years	NR	Higher AdAS scores in all EDs compared to controls. AdAS positively associated with EDI-2 scores, controlling for age and BMI.
			24 AN-BP	100	27.8	NR	14.7 years	NR	
			34 BN	97	32.1	NR	13.7 years	NR	
			34 BED	82	40.5	NR	12.0 years	NR	
			160 control	61	26.5	NR	14.9 years	NR	
Dinkler et al (53)	Longitudinal cohort	Measure Autistic traits in individuals with and without AN, before and after AN onset	5987 general population	52	T1 9 / T2 18^2^	NR	NR	NR	Those with AN showed elevated Autistic traits at age 18 but not at age 9 compared to those who did not develop AN.
Dobrescu et al (61)	Longitudinal case-control	Measure cognitive performance before and after weight restoration in AN, resemblance to their parents’ profiles, and associations with Autistic traits.	20 AN	100	14.2	NR	NR	NR	AQ-50 scores were stable in individuals with AN followed from the acute state to weight recovered (1 year later). Generally, no differences in cognition.
			28 control	100	14.8	NR	NR	NR	
Galvin et al (93)	Cross-sectional survey	Investigate drive for muscularity in the relationship between Autistic traits and EDs	1068 general population	39	28.7	77% White, 8% Asian, 5% Black, 3% Hispanic/ Latino, 2% mixed, 1% Chinese, 1% middle/ near eastern	NR	NR	AQ-50 and EAT-26 scores were significantly positively correlated, controlling for age, sex, and BMI. AQ-50 score was positively correlated with EAT-26 total score independently of anxiety and depression symptoms in females but not males.
Gesi et al (72)	Cross-sectional case-control	Examine whether Autistic traits occur in EDs other than AN	53 AN	NR	NR	NR	NR	NR	Significantly higher AQ, AdAS and RAADS-14 scores were found in all EDs compared to controls. Few differences between EDs.
			41 BN	NR	NR	NR	NR	NR	
			42 BED	NR	NR	NR	NR	NR	
			105 control	NR	NR	NR	NR	NR	
Halls et al (68)^c^	Cross-sectional case-control	Examine whether differences in brain structure are related to Autistic traits in AN	57 AN	100	19.4	NR	NR	NR	AN had significantly higher ADOS-2 communication and social and creativity scores than controls, whereas WR AN had significantly higher stereotyped and repetitive scores than AN and controls. AN showed some differences in gyrification and grey matter compared to WR AN and controls, however these were not related to ADOS-2 scores.
			59 WR AN	100	18.3	NR	NR	NR	
			69 control	100	19.4	NR	NR	NR	
Halls et al (60)^c^	Longitudinal case-control	Examine stability of psychosocial difficulties in AN	94 AN	100	19.1	NR	NR	NR	Three components representing psychosocial difficulties (correlated with EDE-Q, HADS, OCI, and WSAS scores), Autistic traits, and BMI were found. No group difference or change over time (2yrs) in Autistic traits component.
			40 control	100	19.2	NR	NR	NR	
Harrison et al (69)	Cross-sectional case-control	Measure eye contact in acute and recovered AN compared to controls	25 AN	100	27.9	63% White British	16.9 years	NR	Groups did not differ on AQ-50. AN made less eye contact than controls, rec AN between the two.
			25 rec AN	100	26		15.0 years	NR	
			25 controls	100	27		16.7 years	NR	
Huke et al (42)	Longitudinal case-control	Examine Autistic traits in relation to treatment completion and ED psychopathology in AN	32 AN	100	28.7	97% White, 3% mixed White and Caribbean	14.8 years	NR	YBOCS scores were significantly positively associated with AQ-50 scores. BMI, EDE-Q, and HADS depression and anxiety were not.
			32 controls	100	24.9	NR	15.4 years	NR	
Ingrosso et al (76)	Cross-sectional observational	Examine Autistic traits and sensory sensitivity in relation to ED symptoms and autism-related eating behaviour	37 AN	100	25.6	NR	15% middle school, 5% 3-year professional license, 61% diploma, 12% Bachelor's, 7% Master's	NR	Although not tested for significance, more AN participants scored above cut-off on the AQ-50 than the other EDs, whilst on the RAADS-R more BN participants scored above cut-off. The effect of sensory sensitivities on EAT-26 and SWEAA scores was mediated by RAADS-R scores.
			16 BN	100		NR		NR	
			10 BED	100		NR		NR	
			12 OSFED	100		NR		NR	
Kalayci et al (81)	Cross-sectional case-control	Investigate Autistic traits and social responsiveness in AN	39 AN	100	15.2	NR	9.8 years	NR	SRS scores were not correlated with BMI, EAT scores, length of illness, or age at onset. When controlling for BDI, SCARED, and MOCI scores, SRS scores remained significantly higher in AN than controls.
			34 controls	100	14.9	NR	14.9 years	NR	
Karjalainen et al (11)	Longitudinal case-control	Examine eating behaviours associated with autism in those with AN.	36 AN	100	19.6	NR	NR	NR	Significant decrease in AQ-50 and SWEAA total scores in AN between baseline and 1yr later. SWEAA BTSD scores (comprising items that best distinguished between Autistic individuals and controls) were significantly higher in Autistic and AN groups compared to controls and did not change across time.
			19 Autistic	100	18.5	NR	NR	NR	
			30 control	100	18	NR	NR	NR	
Karjalainen et al (36)	Cross-sectional observational	Examine prevalence of EDs and symptoms in ADHD and/or autism.	31 Autistic + ADHD	45	29.5	NR	13% attend compulsory school, 33% attend high school, 47% attend University/ college, 7% have University/ college degree	NR	8 Autistic people (11%) reported a current or previous ED: AN 5 (6.7%); BN 2 (2.7%); BED 1 (1.4%). Those with EDs did not have more comorbid psychiatric disorders than those without.
			35 Autistic	37	32	NR	6% attend compulsory school, 30% attend high school, 39% attend university/ college, 24% have University/ college degree	NR	
			72 ADHD	49	33.4	NR	11% attend compulsory school, 39% attend high school, 44% attend university/ college, 6% have University/ college degree	NR	
Kerr-Gaffney et al (14)^d^	Cross-sectional case-control	Examine utility of SRS-2 as a measure of Autistic traits in AN	49 AN	94	27	NR	16.1 years	NR	AN and rec AN had significantly higher ADOS-2 and SRS-2 scores compared to controls, with no differences between the two. SRS-2 predicted EDE-Q and WSAS scores but were not related to BMI or illness duration.
			49 rec AN	98	26	NR	16.5 years	NR	
			44 controls	91	23.9	NR	16.6 years	NR	
Kerr-Gaffney, Mason, et al (66)^d^	Cross-sectional case-control	Examine emotion recognition in AN	45 AN	94	27	NR	16.1 years	NR	ADOS-2 scores significantly predicted emotion recognition when controlling for group and IQ. AN and rec AN scoring above cut-off were significantly less accurate than those scoring below and controls.
			49 rec AN	98	26	NR	16.5 years	NR	
			46 controls	91	23.9	NR	16.6 years	NR	
Kerr-Gaffney et al (65)^d^	Cross-sectional case-control	Compare Autistic traits in AN, rec AN, Autistic women, and controls	64 AN	100	21.5	NR	NR	NR	On the AQ-10, SRS-2, and ADOS-2, Autistic women generally scored highest, AN and rec AN in the middle, and controls the lowest.
			46 rec AN	100	22.2	NR	NR	NR	
			41 Autistic	100	20.6	NR	NR	NR	
			67 controls	100	22.2	NR	NR	NR	
Kinnaird et al (79)	Cross-sectional case-control	Explore ED symptoms in Autistic versus non-Autistic men	54 Autistic	0	38.8	65% White, 4% Latinx, 2% Mixed, 30% missing	NR	NR	No difference in prevalence of lifetime ED diagnosis or current ED behaviours between groups. Autistic men had higher EDE-Q scores than non-Autistic men.
			49 non-Autistic	0	32.8	80% White, 8% Asian, 2% Latinx, 6% Mixed, 4% missing	NR	NR	
Koch et al (32)	Longitudinal cohort	Investigate co-occurrence of AN and autism in probands and first- and second-degree relatives	1724189 general population (5006 AN and 12606 Autistic probands)	NR general population (93 AN / 21 Autistic)	NR	NR	NR	NR	Individuals with a first diagnosis of autism had increased risk of a later diagnosis of AN, and those with a first diagnosis of AN had increased risk of a later diagnosis of autism. Risk of AN or autism was even greater in those with depression. Co-aggregation of autism and AN in families.
Leppanen et al (89)^c^	Longitudinal cohort	Examine whether Autistic features predict AN symptom profiles across time.	105 AN	100	18.9	90% White European, 7% mixed, 3% Asian	NR	NR	Higher AQ-10 and ADOS-2 scores predicted more severe psychological symptoms across two years, but were not associated with BMI.
Li et al (57)	Longitudinal clinical audit/service evaluation	Evaluate treatment outcomes in individuals with AN with and without Autistic traits.	476 AN	98	27.7	NR	NR	NR	AQ-10 scores significantly decreased from admission to discharge in inpatients, but not daycare or “step-up” patients.
Li et al (88)	Cross-sectional observational	Explore subgroups of AN based on clinical variables using cluster analysis	227 AN	NR	27.5	NR	NR	NR	Two clusters: one with higher levels of psychopathology, AN-BP subtype, higher admission BMI, and higher AQ-10 scores. BMI did not substantially contribute to cluster formation.
Lin et al (55)	Longitudinal case-control	Characterise psychiatric comorbidities in those with and without EDs	14524 ED	79	15.9	69% White, 7% Black / African American, 6% other, 3% Asian	NR	NR	Rates of autism were significantly higher preceding an ED diagnosis than in controls, however, those with EDs were no more likely to receive a subsequent autism diagnosis after an ED diagnosis.
			110051 controls	65	17.8	65% White, 11% Black/ African American, 5% other, 2% Asian	NR	NR	
Mantel et al (90)	Longitudinal cohort	Test association between maternal EDs and offspring neuropsychiatric conditions	4047 Maternal AN	100	29.2^3^	NR	8% <9yrs, 36% 9-12yrs, 54% >12yrs, 2% missing^1^	NR	Increased risk of ADHD and autism among children of mothers with EDs, highest for children of mothers with ongoing AN.
			20235 Unexposed comparator	100	29.2	NR	9% <9 yrs, 41% 9-12 yrs, 48% >12 yrs, 3% missing	NR	
			1185 Maternal BN	100	29.6	NR	8% <9 yrs, 38% 9-12yrs, 53% >12 yrs, 1% missing	NR	
			5925 Unexposed comparator	100	29.6	NR	9% <9 yrs, 37% 9-12yrs, 52% >12 yrs, 3% missing	NR	
			3581 Maternal unspecified ED	100	28.9	NR	11% <9 yrs, 41% 9-12yrs, 46% >12 yrs, 2% missing	NR	
			17905 Unexposed comparator	100	28.9	NR	10% <9 yrs, 40% 9-12yrs, 47% >12 yrs, 3% missing	NR	
McCrossin et al (95)	Cross-sectional modelling study	Explore AN epidemiological data using Bayasian theorem to establish prevalence of autism in females.	General population (various data sets)	100	NR	NR	NR	NR	Estimated prevalence of autism in women in the general population is 6%, 16% in women with mental health conditions, and 25% in women with AN.
Nilsson et al (63)^a^	Longitudinal case-control	Report prevalence of personality disorders, OCD, and autism, 10 years after onset of AN	51 AN	94	24.5	NR	NR	NR	Significantly greater proportion of AN group met criteria for autism than controls (18% vs. 2%) despite 94% being weight restored.
			51 controls	NR	24.2	NR	NR	NR	
Nistico et al (73)	Cross-sectional case-control	To compare ED symptoms and Autistic eating behaviours in EDs, Autistic people, and controls	34 ED	100	30.8	NR	NR	NR	BMI was not associated with EAT-26 or SWEAA scores. ED and Autistic groups had significantly higher SWEAA scores than controls.
			34 Autistic	100	37.8	NR	NR	NR	
			35 controls	100	35.7	NR	NR	NR	
Numata et al (74)	Cross-sectional observational	Explore if self-induced vomiting is associated with Autistic traits in each ED.	23 BN	100	27	NR	NR	NR	No differences in AQ-50 scores between EDs.
			8 AN-BP	100	28.1	NR	NR	NR	
			6 AN-R	100	18.6	NR	NR	NR	
			5 BED	100	28.7	NR	NR	NR	
Nuyttens et al (13)	Longitudinal cohort	Compare Autistic traits in AN-R during and after being underweight	29 AN-R	93	15	NR	NR	NR	AQ-50 scores significantly decreased after weight restoration. BMI change, medication use, and internalising symptoms did not contribute to AQ-50 scores after weight restoration, only baseline AQ-50 scores did.
Parsons (50)	Cross-sectional observational	Define incidence of autism identification in ED patients	40 ED	100	NR	73% White, 25% Hispanic, 3% Asian	NR	NR	10% had an autism diagnosis at admission, and a further 12.5% were given an autism diagnosis during treatment. No difference in Autistic traits between restrictive and non-restrictive EDs.
Pruccoli et al (45)	Cross-sectional observational	Investigate the relationship between Autistic traits, ED psychopathology, and BMI in AN	23 AN	87	15.8	NR	NR	NR	22% scored above cut-off on the ADOS-2, and 9% met cut-offs on both the ADOS-2 and the developmental measure, the AQ 4-11 yrs test. BMI not associated with ADOS-2, AQ-50, or AQ 4-11 yrs scores.
Rødgaard et al (56)	Longitudinal cohort	Investigate how autism comorbidity rates vary according to birth year, sex and age at which autism was first diagnosed	16126 Autistic	26	NR	NR	NR	NR	EDs and other psychiatric disorders were more common in those with a late childhood diagnosis than an early one.
Rastam et al (64)^a^	Longitudinal case-control	Assess outcome of AN over 10 years and the relationship with personality and psychiatric disorders	51 AN	94	24.5	NR	NR	NR	OCD, OCPD, and/or autism continued to characterise >1/3 of the AN group. 1/6 had persistent difficulties with social interaction and obsessive-compulsive behaviours from childhood to early adult years.
			51 controls	NR	24.2	NR	NR	NR	
Rhind et al (82)	Randomised controlled trial	Examine autism spectrum and/ or obsessive-compulsive traits in adolescents with AN and their parents	150 AN or EDNOS-AN	91	16.9	NR	NR	NR	4% met diagnostic criteria for autism. Social aptitude scores were not related to BMI, illness duration, ED symptoms, clinical impairment, anxiety, depression or stress scores.
Schaumberg et al (52)	Longitudinal cohort	Investigate whether task-based (facial emotion recognition) and parent report measures of social communication in childhood associate with ED symptoms and diagnoses in adolescence	4864 general population	NR	8, 10, 14, 16, and 18^2^	NR	NR	NR	SCDC scores during childhood were associated with BN symptoms during adolescence among girls and boys. Misattribution of faces as sad or angry at age 8 was associated with purging and AN diagnosis at age 14 respectively among girls.
Shan et al (77)	Longitudinal cohort	Investigate risk of emotional/behavioural disorders, ADHD, autism, and ID in children with FEDs	1967 FED	53	NR	NR	26% ≤9yrs, 43% 10-15yrs, 31% >15%yrs^1^	NR	Children with FED had increased risk of autism, ADHD, ID, and emotional/behavioural disorders.
			19670 controls	53	NR	NR	19% ≤9yrs, 45% 10-15yrs, 36% >15%yrs	NR	
Socie et al (40)	Cross-sectional observational	Characterise EDs in Autistic children < 4yrs	33 Autistic	18	2.5^4^	NR	65% ≥2 years higher education, 35% ≤2 years higher education^1^	NR	70% children had a parent-reported ED.
Solmi et al (18)	Longitudinal cohort	Compare trajectories of social traits from childhood in adolescents with disordered eating	5381 general population	55	7.6 - 16.8^2^	96% White, 4% ethnic minority	54% compulsory (up to age 16), 46% non-compulsory	NR	Individuals with disordered eating at age 14 had higher SCDC scores from age 7 to mid-adolescence. Disordered eating at age 14 was not associated with higher SCDC scores at age 16 years.
Stewart et al (46)	Longitudinal clinical audit/service evaluation	Impact of Autistic traits on treatment outcomes of girls with EDs	409 ED	100	14.6	NR	NR	NR	AQ-50 scores (current traits) were elevated but DAWBA scores (developmental traits) were not. Both were significantly correlated with parent-reported depression and anxiety and self-reported quality of life.
Susanin et al (59)	Randomised controlled trial	Examine Autistic traits in early stages of AN and throughout treatment to understand whether they are a marker of poor prognosis or sequelae of AN	59 AN	86	15.4	93% White, 98% non-Hispanic	NR	NR	No significant difference in AQ-10 (parent version) from baseline to end of treatment, however, there was a significant reduction in parental API scores.
Tchanturia et al (58)	Longitudinal clinical audit/service evaluation	Examine association between Autistic traits, ED symptoms, and other psychopathology during inpatient treatment in AN	171 AN	100	27.3	NR	NR	NR	Significant decrease in AQ-10 scores from admission to discharge. AQ-10 scores were not related to BMI.
Vagni et al (75)	Cross-sectional observational	Measure Autistic traits in different EDs	29 AN	100	19.8	NR	NR	NR	No association between BMI and AQ-50, RAADS-R, or RAADS-M scores. No difference in frequency of high Autistic traits between EDs. SCL-90 global score explained a significant proportion of variance in RAADS-R but not RAADS-M scores.
			25 BN	100	24.5	NR	NR	NR	
			13 BED	100	27.0	NR	NR	NR	
Westwood et al (87)	Cross-sectional observational	Measure Autistic traits and associations with psychiatric symptoms in AN	60 AN	100	26.6 HAS / 23 SCAS / 22 NAS^4^	NR	NR	NR	No difference in BMI, EDE-Q, HADS, or illness duration between groups with high, subclinical, or no Autistic traits. TAS-20 and OCI-R scores were higher in those with high versus no Autistic traits.
Westwood et al (44)	Cross-sectional observational	Measure current and developmental Autistic traits in AN	40 AN	100	15.2	83% White British, 8% White Other, 5% dual heritage, 3% Indian, 3% Black Caribbean	NR	NR	52.5% scored above the ADOS-2 cut-off, 10% above cut-off on both the ADOS-2 and 3Di-sv, meeting full autism criteria.
Yin et al (78)	Longitudinal cohort	Examine associations between Autistic traits and different ED psychopathology (thinness-oriented, muscularity-oriented, and ARFID)	501 general population	49	31.1	NR	4% high school or below, 96% college or above	56% < 8000 ¥, 44% ≥8000 ¥	In women only, higher Autistic traits at baseline were associated with increased ARFID symptoms 6 months later.
Zenia et al (35)	Longitudinal cohort	Study eating disturbances in children with autism or schizophrenia	90 Autistic	62	Up to 10	NR	NR	NR	High rates of eating difficulties were found throughout the first 10 years of life in both groups.
			77 schizophrenia	66		NR	NR	NR	
Zhang et al (91)	Longitudinal cohort	Explore whether autism in people with EDs is associated with severity, service utilisation, and self-harm	3189 AN	98	NR	NR	NR	NR	Autism diagnosis associated with higher ED severity, remained significant controlling for ADHD and ID. No difference in autism PGS in those with autism+ED diagnosis compared to those with ED only.
Qualitative									
Adamson et al (37)	Interviews	Explore carers’ experiences of treatment for Autistic people with AN	10 carers	90	NR	NR	NR	NR	Carers thought autism played a significant role in the development and maintenance of AN: AN as a coping mechanism for autism-associated difficulties; social difficulties contributing to development of AN; and sensory issues complicating AN.
Babb et al., (38)^d^	Interviews	Explore Autistic women's experiences of ED services from three perspectives (patients, parents, healthcare professionals)	15 Autistic + AN	100	32.6	NR	NR	NR	Autistic participants felt their Autistic traits were misjudged as ED symptoms before they received a diagnosis. HCPs expressed difficulty distinguishing between ED and Autistic behaviours.
			13 parents	NR	NR	NR	NR	NR	
			11 HCPs	NR	NR	NR	NR	NR	
Brede et al (10)^d^	Interviews	Understand how AN develops and persists in Autistic women	15 Autistic + AN	100	32.6	NR	NR	NR	Sensory sensitivities, social and communication difficulties, and specific thinking styles predated ED. Additional mental health difficulties were intertwined with autism and ED.
			13 parents	92	NR	NR	NR	NR	
			16 HCPs	NR	NR	NR	NR	NR	
Creese et al (47)^d^	Secondary data analysis of interviews	Explore the journey to an autism diagnosis in women with co-occurring AN	17 Autistic + AN	100	32.6	NR	NR	NR	Autism had been suspected previously, particularly at school, for most participants however they were not diagnosed until adulthood. Many felt they were misdiagnosed before receiving autism diagnosis.
Doris et al (48)	Interviews	Evaluate friendship experiences in AN with Autistic traits to understand whether they are similar to those of Autistic people and present before ED onset	7 AN	100	24.8	86% white, 14% Afro-Caribbean	NR	NR	Some reported limited friendships in childhood, as well as deterioration of friendships coinciding with ED onset.
Kinnaird et al (39)	Interviews	Explore support needs of carers of Autistic people with AN	11 carers	73	55	NR	NR	NR	Children of carers received autism diagnosis after treatment for other mental health problems, due to not recognising autism or difficulties obtaining a diagnostic assessment. Some thought delayed recognition of autism resulted in later problems.
Li et al (49)	Case discussions	Summarise clinical challenges in supporting Autistic adults with AN	20 AN	80	26	85% white British, 5% white other, 5% black African, 5% Asian.	NR	NR	Care team spent considerable time helping distinguish between issues caused by mental health problems or autism, however these were often intertwined.
Makin et al (41)	Interviews	Explore experiences of BED in neurodivergent individuals	10 ND+BED	90	36	40% White, 20% Black, 20% Asian, 20% mixed	NR	NR	Many were unsure about link between BED and neurodivergence. Described links between food preferences, sensitivities, and fixations and binging.
Case studies/series									
Carmassi et al (30)	Case study	Examine whether undiagnosed autism may have impacted later comorbidities	1 Autistic + BED + BD + PD	100	35	NR	Postgraduate education	NR	Described Autistic traits in childhood, and disordered eating onset in late childhood. Suggested undiagnosed autism may have had a role in response to traumatic events.
Dudova et al (51)	Case study	Present two cases of young Autistic girls with early onset AN	2 Autistic + AN	100	10 and 5	NR	NR	NR	Describes different connections between AN and autism in each patient; in the first both AN and autism diagnostic criteria were fully met, whereas in the latter AN was more a feature of autism.
Mandy & Tchanturia (80)	Case series	Examine whether social and flexibility difficulties seen in EDs are Autistic in origin or only superficially resemble autism.	7 AN-R, 2 EDNOS, 1 BN	100	26.4	NR	NR	NR	All 5 participants who scored above ADOS-2 cut-off had AN-R and were underweight. These participants described long-standing social difficulties predating ED.
Rothery & Garden (31)	Case study	Report case of AN and infantile autism	1 Autistic + AN	100	16	NR	NR	NR	Autism was diagnosed at age 4, but characteristics described earlier, and ED behaviours at age 12.

AN = anorexia nervosa, AN-R = anorexia nervosa, restricting subtype, AN-BP = anorexia nervosa, binge–purge subtype, BN = bulimia nervosa, BED = binge eating disorder, ED = eating disorder, EDNOS = eating disorder not otherwise specified, EDNOS-AN = eating disorder not otherwise specified, anorexia nervosa subtype, OSFED = other specified feeding or eating disorder, ARFID = avoidant/restrictive food intake disorder, FED = feeding and eating disorder, RED = restrictive eating disorder, ND = neurodivergent, BD = bipolar disorder, PD = panic disorder, BPD = borderline personality disorder, OCD = obsessive-compulsive disorder, OCPD = obsessive-compulsive personality disorder, ADHD = attention-deficit/hyperactivity disorder, ID = intellectual disability, IDD = intellectual developmental disorder, HCP = healthcare professional, AQ = Autism-Spectrum Quotient, AQ-10 = 10-item Autism-Spectrum Quotient, AQ-50 = 50-item Autism-Spectrum Quotient, AQ 4–11 yrs = Autism-Spectrum Quotient, child version (4–11 years), ADOS-2 = Autism Diagnostic Observation Schedule, Second Edition, AdAS = Adult Autism Subthreshold Spectrum, RAADS-14 = Ritvo Autism Asperger Diagnostic Scale, 14-item version, RAADS-R = Ritvo Autism Asperger Diagnostic Scale–Revised, RAADS-M = Ritvo Autism Asperger Diagnostic Scale–Modified, SRS = Social Responsiveness Scale, SRS-2 = Social Responsiveness Scale, Second Edition, SCDC = Social and Communication Disorders Checklist, SWEAA = Swedish Eating Assessment for Autism Spectrum Disorders, SWEAA BTSD = Swedish Eating Assessment for Autism Spectrum Disorders, Best Two Subscale Discriminators, EAT-26 = Eating Attitudes Test, 26-item version, EAT-40 = Eating Attitudes Test, 40-item version, EDI-2 = Eating Disorder Inventory-2, EDE-Q = Eating Disorder Examination Questionnaire, HADS = Hospital Anxiety and Depression Scale, BDI = Beck Depression Inventory, SCARED = Screen for Child Anxiety Related Emotional Disorders, MOCI = Maudsley Obsessive-Compulsive Inventory, YBOCS = Yale–Brown Obsessive Compulsive Scale, OCI = Obsessive-Compulsive Inventory, OCI-R = Obsessive-Compulsive Inventory–Revised, WSAS = Work and Social Adjustment Scale, TAS-20 = Toronto Alexithymia Scale, 20-item version, SCL-90 = Symptom Checklist-90, GSQ = Glasgow Sensory Questionnaire, D-Flex-R = Detail and Flexibility Questionnaire–Revised, PGS = polygenic score, API = Autism Probability Index, DAWBA = Development and Wellbeing Assessment, 3Di-sv = Developmental, Dimensional and Diagnostic Interview, short version, IQ = intelligence quotient, BMI = body mass index, NR = not reported, yrs = years, T1 = time point 1, T2 = time point 2, HAS = high Autistic traits subgroup, SCAS = subclinical Autistic traits subgroup, NAS = no Autistic traits subgroup.

Same superscript letters indicate there is some crossover in samples.

^1^Refers to parental/maternal education in this study.

^2^Refers to age at which measures were taken over multiple time points.

^3^Refers to age at delivery in this study.

^4^Median.

### Synthesis of results

#### Secondary impact of EDs

##### Autistic traits or diagnoses in childhood/before ED

Five studies [two with overlapping samples (25, 37)], used a retrospective childhood/developmental measure of autism in individuals with EDs to examine whether Autistic traits were present before ED onset. Westwood et al. (79) found that despite 52.5% adolescents with AN scoring above cut-off on the Autism Diagnostic Observation Schedule Second Edition (ADOS-2), an observational measure of current Autistic traits, only 10% of the sample additionally scored above cut-off on the developmental parent interview, the Developmental, Dimensional and Diagnostic Interview Short Version (3Di-sv), therefore meeting diagnostic criteria for autism. Similarly, Pruccoli et al. (67) reported that 22% of adolescents with AN scored above cut-off on the ADOS-2, whilst 9% met cut-offs on both the ADOS-2 and a developmental measure, the Autism Quotient (AQ) 4–11 years test. Brede (37) and Brede et al. (25) compared Ritvo Autism and Asperger Diagnostic Scale-Revised (RAADS-14) childhood ratio scores in Autistic women without EDs, Autistic women with restrictive EDs, women with restrictive EDs with high Autistic traits but no autism diagnosis, and women with restrictive EDs with low Autistic traits, finding that the Autistic groups and the women with restrictive EDs with high Autistic traits but no autism diagnosis scored significantly higher than the group with restrictive EDs and low Autistic traits. Alongside various other clinical measures, the authors conclude that those with restrictive EDs and high Autistic traits present similarly to those with a formal autism diagnosis. Conversely, Stewart et al. (74) reported that although current Autistic traits (AQ-50 scores) were elevated in adolescents with EDs compared with population norms, early childhood traits, as measured by the Development and Well-Being Assessment (DAWBA), were not.

Six qualitative studies (three with overlapping samples (10, 84, 85);) and three case studies/series also reported childhood Autistic traits in those with EDs. Findings from qualitative studies suggested that Autistic participants with AN felt that autism played a significant role in the development and maintenance of AN, for example, social and sensory difficulties and different thinking styles were felt to be present before AN onset and contributed to the development of the illness (10, 83, 85). However, deterioration of social life alongside illness onset, suggesting starvation effects, was also described (10, 86). Some studies also reported on the timing of AN and autism diagnoses: Adamson et al. (83) reported that 30% of Autistic participants with AN were diagnosed with AN first, Li et al. (88) reported 50% had an autism diagnosis before entering ED services, and Babb et al. (84) reported that none of the Autistic participants with AN had an autism diagnosis before they were first seen by ED services. Rates of autism on entering ED treatment were also reported to be 10% in a retrospective chart review of individuals with mixed EDs, and a further 12.5% were given a diagnosis of autism during treatment (66). Case studies often described the development of EDs in Autistic individuals, who were either diagnosed with autism in childhood or received a late diagnosis, but in both cases Autistic traits were often reported to be contributory to the development of EDs (90, 91, 93).

Four longitudinal studies examined the association between Autistic traits measured in childhood and later disordered eating and/or ED diagnoses in large general population samples. Solmi et al. (18) found that individuals with disordered eating at age 14 had higher Autistic social traits at age 7, throughout childhood and up to mid-adolescence, whereas disordered eating at age 14 was not associated with higher Autistic social traits at age 16 years. Similarly, Carter Leno et al. (17) reported that Autistic traits at age 7 were associated with ED behaviours at age 14, an association that was partially mediated by fussy eating. Findings from Schaumberg et al. (71) were more nuanced: parent-reported social communication difficulties during childhood were associated with BN symptoms during adolescence among girls and boys, however, misattribution of faces as sad or angry (task-based social difficulties) in childhood was associated with purging and AN diagnosis at age 14 respectively, among girls only. Conversely, Dinkler et al. (42) found that those with AN at age 18 showed elevated current Autistic traits in some domains, however they did not show elevated Autistic traits at age 9 compared to those who did not develop AN. Differing results may be due to the low numbers of individuals who developed AN in this study resulting in low power, as opposed to the trait/symptom measures used in the former studies.

Two studies examined risk of EDs in those with a first diagnosis of autism and vice versa in general population samples. Christiansen et al. (40) found that those with a first diagnosis of autism had increased risk for a later ED diagnosis (risk of any ED, AN and eating disorder not otherwise specified [EDNOS] remained significant after controlling for mood and anxiety disorders), and individuals with a first ED diagnosis had increased risk of a subsequent autism diagnosis. Similarly, investigating the association between autism and AN specifically, Koch et al. (56) found that individuals with a first diagnosis of autism had increased risk of a later diagnosis of AN, and those with a first diagnosis of AN had increased risk of a later diagnosis of autism. Using electronic health record data, Lin et al. (60) addressed a somewhat similar question, but compared individuals with EDs to controls without EDs who were initiating antidepressants. In the year preceding first diagnosis of an ED (or antidepressant receipt in the control group), rates of autism were significantly higher in the ED group (8.3%) compared to the control group (2.3%), but this was also the case for several psychiatric diagnoses as well as autism. Conversely, after propensity score matching, whilst those with EDs were more likely to receive a subsequent diagnosis of several psychiatric diagnoses in the year following ED diagnosis, they were no more likely to receive an autism diagnosis. Finally, Rødgaard et al. (68) found that EDs were more common in Autistic individuals who had received a late childhood autism diagnosis than an early one.

In sum, diverse studies assessing Autistic traits or diagnoses in childhood/before ED have shown that (a) approximately 9 - 10% of individuals with AN meet both current and developmental cut-offs for autism; (b) rates of autism diagnoses in people with EDs on entering ED services were highly variable (0 – 70%); (c) autism was described as a contributing factor to the development of EDs, although some Autistic traits worsened with ED onset; and (d) early Autistic traits/diagnoses were linked to later disordered eating/ED diagnoses; and this relationship was sometimes but not always bidirectional.

##### Stability of autistic traits or diagnoses over time, including before and after weight restoration

Seven studies measured Autistic traits in individuals with AN over time, often before and after full or partial weight restoration, to understand whether these traits may be a result of starvation or whether they remain stable despite fluctuations in clinical state. Most studies found a significant decrease in Autistic traits from baseline to follow-up (11, 13, 58, 75, 76), with some caveats. In the largest study examining this question, Li et al. (58) found a significant decrease in AQ-10 scores from admission to discharge in inpatients, but not in patients attending “step-up” or daycare services. Nuyttens et al. (13) reported that AQ-50 scores significantly decreased in individuals with anorexia nervosa restricting type (AN-R) from baseline to follow-up. As this study aimed to assess the impact of weight restoration specifically, those who did not weight restore at follow-up were excluded from the analysis, however, these excluded patients had higher AQ-50 scores at baseline than the rest of the sample. Susanin et al. (75) found that stability of Autistic traits differed by scale used: parental AQ-10 scores did not change but parental Autism Probability Index (API) scores significantly decreased from baseline to end of treatment 6 months later in adolescents with AN. It must be noted that this study excluded individuals with an autism diagnosis, which seriously limits the validity of the findings, given one would expect those with a diagnosis of autism to have relatively stable scores across time. Finally, two studies reported no significant change in Autistic traits across time in individuals with AN, one following acute AN one year later when they had weight restored, and the other following a group of individuals in mixed states (recovered and acute AN) two years later (43, 47).

Three papers reporting on the same longitudinal study following a sample of individuals with AN up to 18 years after onset reported the stability autism diagnoses across time (31, 63, 69). At the fourth wave of the study, three of the 51 participants still had AN and the rest were normal weight (three of which had a persisting ED). Six (11.8%) met diagnostic criteria for autism at all four waves of the study, whilst a further 10 (19.6%) met criteria at one to three timepoints. In comparison, only one participant in the control group (2.0%) received an autism diagnosis and this was considered secondary to a substance use disorder. Agreement of autism diagnoses across the waves ranged from k = 0.70 to 0.80, although agreement with premorbid diagnoses was k = 0.23. In sum, studies using self-report Autistic trait measures, which are likely influenced by state effects, show reductions in scores with full or partial weight-restoration. However, studies examining autism diagnoses across time show a significant proportion of individuals with AN (~12%) meet diagnostic criteria across an 18-year period.

##### Autistic traits or diagnoses in acute and recovered AN

Seven studies (three with overlapping samples (14, 53, 54);) investigated a similar question but with a between-subjects design, comparing Autistic traits between individuals with acute AN and those who had recovered or weight restored. Generally, these studies found elevated Autistic traits in both acute and recovered AN compared to controls, with few differences between the two (14, 34, 46, 53, 54). However, one study found no differences in AQ-50 scores between acute AN, recovered AN, and controls (48), whilst another found that those with acute AN had higher parental-rated Autistic traits than those with a history of AN at age 18, who did not differ from those without a history of AN (42). Kerr-Gaffney et al. (54) also included a group of Autistic women, finding that across measures of Autistic traits (AQ-10, social responsiveness scale, 2^nd^ edition [SRS-2], and ADOS-2), Autistic women generally showed the highest scores, controls the lowest, and individuals with AN and those recovered from AN scored between the two. However, like Susanin et al. (75), this study excluded individuals with AN or recovered from AN who had an existing autism diagnosis (n = 6, 5%). This may have led to underestimation of Autistic traits in these groups.

##### Autism and other EDs

Examining whether elevated Autistic traits or diagnoses are present in people with EDs that are not typically associated with being underweight can also help delineate whether Autistic traits are a result of starvation or reflect a transdiagnostic feature across EDs. Almost all studies that included individuals with EDs other than AN reported elevated Autistic traits or diagnoses, similar to those with AN (32, 41, 45, 64–66, 77, 90), although there were some differences depending on the measure of Autistic traits used in one study (50). One qualitative study also reported experiences of BED in neurodivergent individuals (including individuals with suspected autism), describing interactions between Autistic traits (e.g., sensory seeking) and ED behaviours (e.g., binge eating) (89). However, participants generally felt unsure of the link between neurodivergence and BED, as this hadn’t been discussed with them by clinicians.

Additionally, longitudinal studies in population samples have suggested an association between other EDs and autism. As previously discussed, Christiansen et al. (40) reported that individuals with a first diagnosis of autism had significantly greater risk of any ED, AN, and EDNOS, but not BN after controlling for mood and anxiety disorders, whereas Shan et al. (72) reported that those with a childhood feeding and eating disorder before age 3 had increased risk of autism diagnosis, ADHD, ID, and emotional/behavioural disorders. One study reported mixed findings depending on gender: in men, there was no association between Autistic traits at baseline and ED psychopathology at 6 months later, however, in women, higher Autistic traits at baseline were significantly associated with greater ARFID symptoms 6 months later (but not thinness- or muscularity-oriented ED psychopathology) (80).

Several studies examined other EDs or ED symptoms in Autistic individuals, finding that ED and their symptoms tended to co-occur in Autistic individuals. Two studies reported high rates of childhood feeding and eating problems in Autistic children (including lack of appetite, difficulties with swallowing, and food selectivity) (73, 81). Bertelli et al. (35) found that Autistic individuals with ID/IDD showed higher prevalence of feeding and eating disorders than those with ID/IDD only, in particular, BN, pica, food refusal, and food selectivity, despite no difference in BMI between groups. Karjalainen et al. (52) found that 11% of Autistic individuals reported a current or previous ED (6.7% AN, 2.7% BN, and 1.4% BED). Although not tested for statistical significance, rates of AN and BN appeared higher in Autistic individuals than those with ADHD, and rates of BED lower. Regarding ED symptoms, these tended to focus less on calories and body dissatisfaction in Autistic individuals than those with ADHD.

Finally, two studies did not find an association between other EDs and autism. Kinnaird et al. (55) reported no difference in prevalence of lifetime EDs or current ED behaviours between Autistic and non-Autistic men, although Autistic men did have higher levels of ED psychopathology. Mandy and Tchanturia (92) reported a case series of 10 women (7 with AN-R, 2 with EDNOS, and 1 with BN) attending ED services who were queried by their care teams as possibly being Autistic. The ADOS-2 was conducted, and it was found that 50% of the women scored above cut-off, all of whom had AN-R, however this study is limited by the small number of participants and selective sampling.

Although there has been much less research into autism and EDs other than AN, results from these studies suggest (a) elevated Autistic traits in other EDs; (b) a greater risk of some EDs in those with a first diagnosis of autism; (c) an association between childhood feeding and eating disorders and autism; and (d) some suggestion that the connection between autism and other EDs may be weaker in males than in females.

##### Associations with BMI

All included studies that examined cross-sectional associations between BMI and Autistic traits in individuals with EDs found no significant association between the two (14, 25, 34, 36–38, 49, 51, 64, 67, 70, 76, 78). A few studies also included illness duration, again finding no association with Autistic traits (14, 36, 49, 78). One study used cluster analysis to explore subgroups of patients with AN based on clinical variables and the relationships between them (59). Two clusters were found, with one of the clusters showing higher levels of psychopathology, binge-purging subtype, higher admission BMI, and higher Autistic traits. However, BMI did not make substantial contributions to cluster formation, unlike the other variables. Similarly, using principal component analysis, Leppanen et al. (57) demonstrated that higher baseline Autistic traits were associated with worse psychological symptoms but not BMI up to two years later.

##### Genetics of autism and EDs

Four studies examined genetics of autism and EDs. Both Christiansen et al. (40) and Koch et al. (56) reported co-aggregation of EDs and autism within families. Additionally, Christiansen et al. (40) reported that individuals with AN had higher autism polygenic scores than controls, whereas Autistic individuals did not differ in AN polygenic scores compared to controls. Similarly, Mantel et al. (61) reported increased risk of autism among children of mothers with EDs, regardless of subtype, compared with children of mothers with no ED. Increased risk was highest for children of mothers with current AN. Finally, Zhang et al. (82) examined whether autism (diagnosis or polygenic scores) in individuals with EDs was associated with ED severity, service utilisation, or self-harm. Autism polygenic risk scores were not significantly greater in those with an autism and ED diagnosis compared to those with an ED only. Further, whereas an autism diagnosis was associated with a more severe ED on almost all investigated indicators, autism polygenic score was not.

#### Impact of co-occurring conditions

Several studies statistically controlled for co-occurring conditions (including anxiety, depression, OCD, internalising symptoms, trauma, general psychopathology, ADHD, and ID) in analyses investigating the association between EDs and autism, most of which found that the association was not fully accounted for by comorbidities (13, 33, 37, 39, 40, 44, 51, 77, 82). However, two studies found evidence to suggest the association between EDs, and autism was accounted for by co-occurring conditions. In a student sample, Acikel and Cikili (30) found that the relationship between ED symptoms and Autistic traits became non-significant when OCD symptoms were controlled for. Calderoni et al. (38) compared Autistic traits in individuals with AN and controls with and without internalising symptoms, finding that both AN and controls with internalising symptoms scored similarly on the AQ-50, higher than controls without internalising symptoms, suggesting AQ-50 scores may be inflated by general internalising psychopathology.

Five studies investigated whether the association between autism and EDs is non-specific, i.e., whether risk of autism is of similar magnitude to other psychiatric disorders. Koch et al. (56) found that those with AN had increased risk of later autism and that Autistic individuals had increased risk of later AN, however, increased risk of autism or AN was even higher for those with an initial diagnosis of depression. Similarly, although family history of AN was associated with a diagnosis of autism and vice versa, this risk was of the same magnitude as those seen in families with a history of depression or any psychiatric disorder. As previously discussed, Lin et al. (60) found significantly greater rates of autism in people later diagnosed with an ED than controls initiating antidepressants, however this was also the case for mood disorders, generalised anxiety disorder (GAD), ADHD, post-traumatic stress disorder (PTSD), social phobia, and personality disorders, as well as autism. Conversely, individuals with EDs were more likely to receive a subsequent diagnosis of a mood disorder, GAD, PTSD, personality disorder, or phobia, but not autism or ADHD. Rødgaard et al. (68) reported that EDs were more common in individuals who received a late childhood autism diagnosis than an early one. This was also the case for affective disorders, anxiety disorders, OCD, and psychotic disorders, possibly suggesting that undiagnosed autism may contribute to issues that could have been prevented had autism been recognised earlier. Shan et al. (72) reported that children diagnosed with a feeding and eating disorder before age 3 had increased risk of autism, ADHD, ID, and childhood emotional and behavioural disorders. Finally, using existing epidemiological data and Bayes’ theorem, McCrossin (62) estimated the prevalence of autism in women in the general population to be 6%, 16% in women with mental health conditions, and 25% in women with AN specifically, suggesting the link between autism and AN may be greater than that of other mental health conditions.

Three qualitative studies and one case study explored connections between autism, EDs, and other conditions. Brede et al. (10) reported that almost all Autistic women with AN interviewed described additional mental health difficulties that were intertwined with their autism and ED. In interviews with carers of Autistic people with AN, all participants reported that their child only received an autism diagnosis after receiving treatment for other mental health problems (most commonly AN), either due to non-recognition of autism or difficulties with getting a diagnostic assessment (87). They also felt that delayed recognition of autism resulted in later mental health problems. In a qualitative analysis of case discussions of 20 patients with AN, Li et al. (88) reported that clinical teams spent considerable time helping patients distinguish between problems caused by different comorbidities or autism, but these were often intertwined, sometimes fuelling one another. A case report by Carmassi et al. (90) described an individual with BED, bipolar disorder, and panic disorder who received a late diagnosis of autism. The woman also experienced abuse in childhood, and the authors suggest that late diagnosis of autism may have contributed to additional mental health issues.

In sum, studies exploring autism, EDs, and co-occurring conditions suggest that (a) the association between autism and EDs is not fully accounted for by co-occurring conditions, (b) the association is likely non-specific, i.e., autism is also associated with increased likelihood of other conditions; and (c) delayed recognition of autism may contribute to mental health problems.

## Discussion

The aim of this review was to evaluate existing evidence on the role of acute illness effects and co-occurring mental health difficulties in the relationship between autism and EDs. Although evidence was mixed with regards to the role of starvation in those with restrictive EDs, longitudinal and qualitative evidence reporting Autistic traits in childhood, a lack of association between BMI and Autistic traits, and a link between autism and EDs not typically associated with low body weight suggests that the association between autism and EDs is not solely due to acute illness effects. Similarly, although high rates of co-occurring mental health problems were reported in those with EDs, these were not found to fully account for co-occurring autism or Autistic traits. Longitudinal and genetic evidence supported a general increased risk of mental health problems in Autistic individuals, complemented by qualitative evidence expanding on the interrelations between autism, EDs, and other mental health conditions.

Studies in those with restrictive EDs suggest that elevated Autistic traits are a result of a combination of state and trait effects. Several studies measuring Autistic traits over time in people with AN, often before and after individuals were partially or fully weight-restored, showed significant reductions in Autistic traits from baseline to follow-up (11, 13, 58, 75, 76). However, this was not the case for all studies, or subgroups within studies (43, 47, 58). Further, cross-sectional studies comparing levels of Autistic traits in people with current AN to those who have recovered generally showed elevated traits in both groups, with few differences between the two (14, 34, 46, 53, 54). Concurrently, a lack of any association between Autistic traits and BMI or illness duration was found. These results might suggest that although Autistic traits may be elevated in the acute state of illness and may reduce in some individuals with weight restoration when measured at the group level, there is a significant proportion that continue to display high Autistic traits after recovery. This interpretation is supported by the only study measuring stability of autism diagnoses across time in AN, finding that although approximately 20% of the sample met diagnostic criteria at one to three waves of the study (suggesting possible fluctuating state effects), approximately 12% of the sample met diagnostic criteria at all four waves of the study (suggesting an Autistic subgroup/trait effects) (31, 63, 69).

Further evidence for a combination of state and trait effects comes from studies utilising both current and developmental assessments of Autistic traits, although very few studies did so. The prevalence of clinically significant current Autistic traits was reported to be as high as 52.5% in individuals with AN (79). However, the proportion meeting both current and developmental trait cut-offs was reported to be 9 and 10% in the two studies that this data (67, 79), very similar to the figure reported in the longitudinal study by Anckarsäter et al. (31). Thus, whilst measuring Autistic traits on the group level picks up both those who are Autistic and those who may be showing Autistic behaviours as a result of starvation or other illness effects, using developmental measures or repeated assessments over time shows that approximately 9 - 12% of individuals with AN also meet diagnostic criteria for autism. This figure is substantially greater than the prevalence in the general population (0.9%), especially in women (0.2%) (94).

Studies with diverse methodologies reported an association between early Autistic traits or diagnoses and later EDs, with some also suggesting a role for autism in the development of EDs and other mental health conditions. Longitudinal studies generally supported a relationship between early autism diagnosis or traits and later ED or ED symptoms, which provides evidence against starvation or illness effects as a sole explanation for Autistic traits, as well as tentatively suggesting the role of autism in the development of EDs (17, 18, 40, 56, 60, 71, 80). This was echoed in qualitative research in which Autistic individuals with AN and their carers explained the ways in which Autistic traits may have contributed to the development and maintenance of their ED (10, 83). However, the reverse was also reported - an increased risk of autism diagnosis in those with an initial ED diagnosis (40, 56). There are a number of possible explanations for this finding, for example, Autistic individuals with EDs often report that autism was only recognised and diagnosed after receiving treatment for other mental health conditions (85, 87). This may be due to a lack of awareness of the way in which autism presents in women (who are at increased risk of EDs compared with men), as well as diagnostic overshadowing (95). Relatedly, two studies suggested that receiving a late autism diagnosis may contribute to the development of EDs and other mental health conditions (68, 87). These findings underscore the importance of improving early identification of autism, especially in women, so that support can be given in childhood and adolescence, key periods for the development of other mental health issues (96).

There was little evidence to suggest that comorbid mental health conditions in those with EDs accounted for high Autistic traits or diagnoses. At the same time, qualitative research described ways in which other mental health conditions interacted with ED symptoms and autism, making it difficult to distinguish between behaviours driven by autism or mental health problems (10, 84, 88), sometimes leading to misdiagnosis (85). Some genetic and longitudinal evidence using large, population-based samples suggested that the association between EDs and autism was non-specific, i.e., autism is also associated with increased risk of other mental health problems (56, 60, 68, 72). This is consistent with past research showing increased risk of all mental health problems in Autistic individuals compared with the general population (97). Together, these findings emphasise an urgent need for autism-informed mental health care across all mental health services, not just ED services. Although autism-adapted therapeutic protocols for anxiety and depression exist (e.g (98, 99).,), these have not yet been implemented within NHS services, and the same CBT-based psychological therapies are recommended for both Autistic and non-Autistic people (100), with questionable efficacy (101–103). Furthermore, staff working within general mental health services often report a lack of knowledge and confidence in working with Autistic people (104). Embedding autism training and adapted interventions across services is needed to address this gap in healthcare provision. This may include, for example, adapted environments to reduce sensory stress, using clear communication and avoiding ambiguous language, providing written or visual material and agendas, and allowing more time for building rapport in psychological therapies.

Strengths of this review include the extensive scope of the literature, including diverse study designs, and consideration of multiple areas of concern that have been highlighted as potential limitations to autism and ED research (8, 9, 23). However, several weaknesses should be noted. Studies were very heterogeneous in design and aims, making synthesis of results and cross-study comparison challenging. Studies often did not report key demographic or socio-economic characteristics, such as race or ethnicity or level of education. Those that did included overwhelmingly white and highly-educated samples, which limits the applicability of our findings to other ethnic/racial groups and socio-economic classes. EDs are reported to affect other ethnicities to a similar degree as White individuals (105), however ED research, as well as healthcare research more generally has lacked both ethnic and socio-economic representation (106). This has resulted in, for example, assessment tools which do not adequately capture the full range of ED behaviours or cognitions (e.g., those arising from food insecurity or differences in beauty ideals) (107). Possible ways in which this could be improved include enhancing participant-researcher concordance by employing diverse research staff to improve trust and participation.

A further limitation is that due to the scoping nature of this review, a critical appraisal of study design was not included. Study limitations that may reduce our confidence in the results include small sample sizes in some studies and likely inadequate statistical power, exclusion of participants with autism diagnoses when investigating Autistic traits in ED populations, completer analysis designs (i.e., excluding participants that dropped out from treatment), a reliance on cross-sectional designs, and trait-based autism measures rather than diagnostic tools. Self-report measures of Autistic traits focus on a narrow set of traits at one point in time, which may fail to capture the full spectrum of Autistic characteristics across the lifespan, especially in women (108). Further, specific self-report measures such as the AQ-10, used in several studies included in this review, have been found to have unsatisfactory test-retest reliability (109), and to poorly predict autism diagnosis (110).

Review findings highlight several important avenues for further research. The majority of autism and ED research uses traits as opposed to diagnosed Autistic samples, which has been highlighted previously (7). While similar outcomes and clinical characteristics have been reported in individuals with EDs with high traits Autistic traits versus a diagnosis (25), future research should endeavour to shift away from the current over-reliance on Autistic traits in ED populations and begin to explore ED symptoms or diagnosis in Autistic populations. This review also highlights a lack of research examining autism and EDs other than AN. Studies included in this review do support elevated co-occurrence of autism and other EDs, however considerably less is known about outcomes, experiences, and treatment of Autistic people with these EDs. Further research is needed to build on this sparse evidence base, with a view to improving awareness, identification and access to support for these presentations. For example, studies could inform training on autism and EDs for healthcare professionals, or the development of improved treatment guidelines. A few studies also highlighted a role for timing of autism diagnosis, suggesting an early diagnosis may prevent later development of EDs, a finding that has been reported previously (111). These findings highlight the importance of expanding research into early identification of autism, especially in women. Relatedly, research into the effectiveness of initiatives that foster positive mental health in Autistic young people is also required. Expanding research in these areas will make important contributions to improving evidence-based practice and improve outcomes for Autistic people.

This review presents an overview of research investigating the overlap between autism and EDs, with a focus on delineating the secondary effects of EDs (e.g., starvation) and co-occurring mental health conditions. Findings from studies with diverse methodologies suggest that elevated Autistic traits and diagnoses in ED populations are not a result of the secondary effects of EDs, although they may be contributory in AN. Similarly, co-occurring mental health problems did not fully explain autism diagnoses or traits in individuals with EDs, although evidence suggests the link between autism and EDs may also apply to other mental health problems. Limited but tentatively supportive genetic evidence of a link between autism and EDs was also found. Several areas for further research are highlighted, most notably a shift away from measuring traits in ED populations and towards investigating outcomes and experiences of EDs in Autistic people, and a need for research into EDs other than AN. Clinically, the research highlights an aetiological link between autism and EDs, which must be recognised in treatment and prevention efforts. Such recognition would require training and awareness in schools, primary health care, mental health services, and in the wider public. More widely, the review highlights a need for early identification and support for Autistic individuals in childhood and adolescence to foster positive mental health and development.

## Data Availability

The original contributions presented in the study are included in the article. Further inquiries can be directed to the corresponding author.
